# Black Beans, Fiber, and Antioxidant Capacity Pilot Study: Examination of Whole Foods *vs.* Functional Components on Postprandial Metabolic, Oxidative Stress, and Inflammation in Adults with Metabolic Syndrome

**DOI:** 10.3390/nu7085273

**Published:** 2015-07-27

**Authors:** Elizabeth J. Reverri, Jody M. Randolph, Francene M. Steinberg, C. Tissa Kappagoda, Indika Edirisinghe, Britt M. Burton-Freeman

**Affiliations:** 1 Department of Nutrition, University of California, Davis, One Shields Avenue, Davis, CA 95616, USA; E-Mails: ebjordan@ucdavis.edu (E.J.R.); jmrandolph@ucdavis.edu (J.M.R.); fmsteinberg@ucdavis.edu (F.M.S.); 2 Departments of Cardiovascular Medicine, Cardiology, and Internal Medicine, Lawrence J. Ellis Ambulatory Care Center, University of California, Davis Health System, 4680 Y Street, Suite 0200, Sacramento, CA 95817, USA; 3 Center for Nutrition Research, Institute for Food Safety and Health, Illinois Institute of Technology, 6502 South Archer Road, Bedford Park, IL 60501, USA; E-Mail: iedirisi@iit.edu

**Keywords:** postprandial, bean, fiber, antioxidant capacity, metabolic syndrome

## Abstract

Beans (*Phaseolus vulgaris*) contain bioactive components with functional properties that may modify cardiovascular risk. The aims of this pilot study were to evaluate the ability of black beans to attenuate postprandial metabolic, oxidative stress, and inflammatory responses and determine relative contribution of dietary fiber and antioxidant capacity of beans to the overall effect. In this randomized, controlled, crossover trial, 12 adults with metabolic syndrome (MetS) consumed one of three meals (black bean (BB), fiber matched (FM), and antioxidant capacity matched (AM)) on three occasions that included blood collection before (fasting) and five hours postprandially. Insulin was lower after the BB meal, compared to the FM or AM meals (*p* < 0.0001). A significant meal × time interaction was observed for plasma antioxidant capacity (*p* = 0.002) revealing differences over time: AM > BB > FM. Oxidized LDL (oxLDL) was not different by meal, although a trend for declining oxLDL was observed after the BB and AM meals at five hours compared to the FM meal. Triglycerides and interleukin-6 (IL-6) increased in response to meals (*p* < 0.0001). Inclusion of black beans with a typical Western-style meal attenuates postprandial insulin and moderately enhances postprandial antioxidant endpoints in adults with MetS, which could only be partly explained by fiber content and properties of antioxidant capacity.

## 1. Introduction

Diet and lifestyle patterns play a significant role in the development of Metabolic Syndrome (MetS) and the diseases it precipitates, namely cardiovascular disease (CVD) and type 2 diabetes mellitus [1]. In particular, MetS development is associated with the consumption of foods typical of the Western diet, including red meat and fried foods [2]. The Western diet is characterized by excess intake of energy, readily available carbohydrates and fats that result in postprandial hyperglycemia and hyperlipidemia [3]. Three large scale prospective cohort studies support the relationship of postprandial lipemia and risk of cardiovascular disease [4,5,6]. Postprandial inflammation and oxidative stress are postulated to be products of hyperglycemia and lipemia and are associated with impaired insulin sensitivity. Impaired insulin responses to Western meals exaggerate postprandial hyperglycemia and lipemia, creating a vicious cycle leading to vascular dysfunction and damage that, over time, likely augments risk for CVD [3].

Reducing transient fluctuations of these postprandial (fed) responses is one lifestyle modification for the prevention and management of MetS [7]. The addition of certain foods to the diet may attenuate these postprandial responses. Foods rich in dietary fiber have been shown to modulate postprandial lipemia [7] and aid in glycemic control [8]. Antioxidant-rich foods have been shown to attenuate postprandial oxidative status markers [9] and increase plasma antioxidant capacity [7]. Beans are a food that provide both dietary fiber and possess high antioxidant capacity [10].

Few studies have investigated the postprandial response to beans (*Phaseolus vulgaris*) [11,12,13,14,15,16] and only one has studied black beans [16]. These few studies have demonstrated that different types of beans elicit different biological responses [17]. This may be in part due to dosing differences, polyphenol bioavailability, and endogenous factors [18]. For example, polyphenols modulate digestive enzymes, eliciting beneficial effects on the postprandial responses. Anthocyanidins in particular, which are found in black beans, have been shown to inhibit α–amylase, maltase, and sucrase activity, which lower postprandial blood glucose [19]. Further, metabolic health benefits of beans are likely related to the functional effects of their inherent dietary fiber and polyphenol content [20,21], the latter being questioned as an effect of the general antioxidant properties of polyphenols or a specific effect of the polyphenol structure. Nowadays, it is popular to isolate and sell functional components of foods as dietary supplements and many supplements are marketed for their “antioxidant” properties. However, functional ingredients may not produce the same effects when delivered outside a whole food matrix [10]. Further, delivery of health benefits based on antioxidant capacity has come under intense scrutiny in recent years [22].

Therefore, we studied the effects of beans on postprandial metabolic, oxidative stress, and inflammatory responses with the focus of understanding the contribution of food matrix and two specific properties: fiber and antioxidant capacity on biological responses in adults with MetS. We hypothesized that meals with whole black beans would produce greater benefits on postprandial responses than meals with added fiber or added antioxidant capacity.

## 2. Experimental Section

### 2.1. Study Participants

The UC Davis Institutional Review Board approved the study and the study was registered at clinicaltrials.gov identifier NCT01190384. The study was conducted at the Ragle Human Nutrition Research Center on the UC Davis campus. Participants were recruited from the greater Sacramento area via flyers and websites. Interested participants were screened by phone for inclusionary and exclusionary criteria. Eligible participants were required to be adults ages 18 and older and meet criteria for MetS [23].

Exclusionary criteria included diagnosis of a chronic disease (such as diabetes mellitus), known allergies/intolerances to study foods, dieting, unusual dietary habits (such as pica), pregnancy or lactation, smokers, drug or alcohol addiction and in recovery <1 year, excessively exercising, on medications that interfered with outcome measurements as assessed by the study physician, or reported difficulty with phlebotomy or an indwelling catheter. Participants who qualified after the phone screening were invited for in-person screening to provide written informed consent and confirm MetS with a fasting blood draw and anthropometric measurements.

### 2.2. Study Design

The study design of this pilot study was a randomized, controlled, crossover trial. There were a total of three study days, each separated by a one week washout period. Consistent with suggested postprandial methods [7], participants were instructed to consume the same meals, refrain from alcohol consumption, and avoid excessive exercise on the day before each study day. After a 12 hour overnight fast, participants arrived at the research center. The study registered nurse inserted an indwelling catheter into the antecubital vein and blood draws were obtained at baseline (fasting) and postprandially every hour for five hours for a total of six blood draws per study day. Five hours was chosen to extend the findings of previous bean trials that assessed postprandial metabolic and inflammation markers after three hours [12,14,16] and represent the usual amount of time between meals [8]. After the baseline blood draw, a moderate-fat breakfast was provided to participants, which included one of three experimental soups to create the black bean (BB) meal, fiber matched (FM) meal, or antioxidant capacity matched (AM) meal. Meal consumption occurred within 20 min and was monitored to ensure compliance. Thereafter, bottled water was provided to the participants *ad libitum*.

### 2.3. Meal Composition

The moderate-fat breakfast consisted of commercially available foods typical of a Western-style breakfast and these foods were prepared as previously described [9]. Soups were provided as part of the meal and a Registered Dietitian matched for macronutrients using nutrition software and nutrition facts labels and for antioxidant capacity using the oxygen radical absorbance capacity (ORAC) method [9,24].

The BB meal consisted of the moderate-fat meal and a soup made from dried black beans that was prepared according to the package instructions and carried dietary fiber and antioxidant capacity. The FM and AM meals comprised of the moderate-fat meal and soups that were couscous-based and supplemented with Instantized BiPro, a whey protein isolate (Davisco Food International, Eden Prairie, MN, USA), to match the protein level of the BB soup.

Additionally, the FM soup was supplemented with Unifiber, an insoluble fiber supplement made mostly of powdered cellulose, but also included some maltodextrin and xanthan gum (DrNatura, Fort Mill, SC, USA), and a 100% pure psyllium fiber supplement (Konsyl, Easton, MD, USA), to match the soluble and insoluble fiber content of the BB soup, but lack the antioxidant capacity. Specifically, the FM soup was matched for the soluble and insoluble dietary fiber of black beans, as the detailed chemical composition of black bean cell walls is currently unknown and, if it were known, it would be difficult to extract [25]. The AM soup was supplemented with 300 mg grape seed extract (GSE) (Polyphenolics, Madera, CA, USA) that was added prior to consumption to match the antioxidant capacity of the BB soup, but lack the fiber content. Each moderate-fat breakfast and soup (BB, FM, or AM) provided an average amount of fat in a typical meal [7] and the soups alone comprised ~30% of the calories of the entire breakfast (Table 1).

**Table 1:** Meal Composition.

Nutrient	Units	BB Meal	FM Meal	AM Meal
Calories	kj	3845	3904	3862
	kcals	919	933	923
Fat	g	25	25	25
Saturated fat	g	10	10	10
Monounsaturated fat	g	4	4	4
Polyunsaturated fat	g	1	1	1
Carbohydrate	g	129	135	133
Fiber	g	18	17	4
Insoluble fiber	g	4	2	0
Soluble fiber	g	10	8	0
Protein	g	42	39	39
Total ORAC	μmol/L trolox equivalents	4499	580	5758

BB, black bean; FM, fiber matched; AM, antioxidant capacity matched; ORAC, oxygen radical absorbance capacity.

### 2.4. Food Records

Weighed food records were obtained three days prior to each study day for a total of nine days of food records. Participants were provided an electronic food scale and encouraged to submit nutrition facts labels. The food records were analyzed by nutrition software (Food Processor SQL Version 10.2, esha Research, Salem, OR, USA).

### 2.5. Biochemical Measurements

On each study day, ~90 mL of blood was drawn into EDTA blood collection tubes and immediately placed onto ice. After ~30 min, blood samples were centrifuged at 1800 X g for 15 min at 4 °C to obtain plasma and frozen at −80 °C until lab analyses were performed in-batch. Biochemical measurements were performed according to manufacturers’ instructions, including appropriate quality controls.

Triglyceride and glucose concentrations were analyzed by enzymatic colorimetric assays using a clinical autoanalyzer (Randox Laboratories, Kearneysville, WV, USA). High sensitivity C-reactive protein (hs-CRP) was analyzed by a chemiluminescent immunometric assay (Fisher Scientific, Pittsburgh, PA, USA). Pro-inflammatory cytokines, interleukin (IL)-6 and IL-1β, and biomarkers of vascular endothelial activation, soluble intercellular adhesion molecule1 (sICAM1) and soluble vascular cell adhesion molecule1 (sVCAM1), were measured by ELISA (R&D Systems, Minneapolis, MN, USA). Insulin was analyzed by an AlphaLISA assay (PerkinElmer, Waltham, MA, USA). Insulin resistance and sensitivity were calculated using homeostasis model assessment (HOMA)-insulin resistance, HOMA-β cell function [26], quantitative insulin sensitivity check index [27], and fasting glucose:insulin.

Oxidized LDL (OxLDL) was also analyzed by ELISA (Mercodia, Winston Salem, NC, USA). Intra- and inter-variability for IL-6 were 5.7% and 16.4%, sICAM1 7.4% and 9.5%, sVCAM1 3.3% and 10.1%, and OxLDL 4.5% and 5.4%, respectively. Curve-fitting and interpolation of ELISA values were obtained by Graphpad 4.03 (Graphpad Software, La Jolla, CA, USA). Plasma hydrophilic and lipophilic Oxygen radical absorbance capacity (ORAC) values were measured according to Prior *et al.* [24] with minor modifications [9].

### 2.6. Statistical Analysis

Outcome measurements were postprandial lipemia, inflammatory biomarkers, oxidative status, and glycemic control responses. Variables not meeting normality and/or homogeneity of variances assumptions were transformed using a log transformation. Data were analyzed by analysis of variance using a mixed model (SAS Version 9.3, Cary, NC, USA). Baseline concentrations of biochemical measurements were included in the model as a covariate when significantly different at baseline. Main effects were analyzed as meal, time, and meal × time interactions. Random effects included participants and meal order. Post-hoc comparisons used the Bonferroni adjustment to correct for multiple comparisons. Statistical significance was considered at *p* < 0.006 and data was presented as least squares means (LSM) as an estimate of the five hour postprandial response after the BB, FM, and AM meals or mean ± SD.

As a pilot study, the recruitment goal was 12 participants. A power analysis revealed adequate power to detect significant differences with >80% confidence for IL-6, sVCAM1, insulin, and total ORAC using a sample size of *n* = 12 per meal, type I error rate of 0.05, observed differences (0.4, 42.4, 0.12, and 0.15, respectively), and observed variability (0.3, 30.9, 3.2, and 4.3, respectively).

## 3. Results

**Figure 1:**
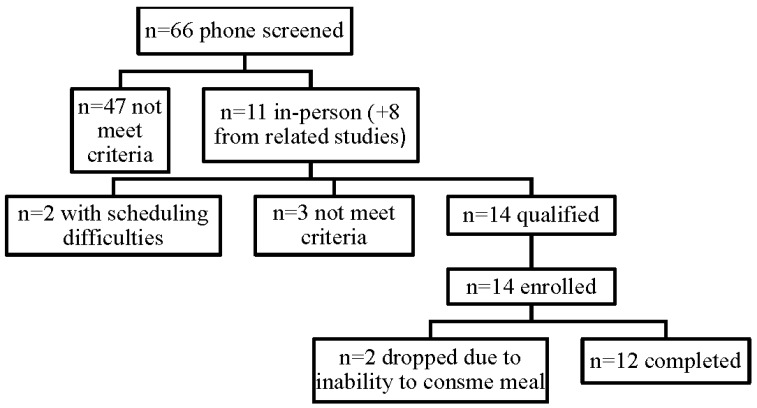
Participant Flow.

Fourteen (*n* = 14) participants were enrolled in the study, two participants were dropped from the study due to inability to complete the breakfast within the allotted time, and twelve participants completed the study (Figure 1). All participants met the criteria for MetS [23]. Half (50%) of the participants were Caucasian, 25% Hispanic, and 25% Asian. The mean age of participants was 49 ± 14 years and BMI 32.2 ± 5.7 kg/m^2^. Half (50%) of the participants were women and all presented with evidence of insulin resistance at baseline (Table 2) [26,27].

The weighed food records obtained three days prior to each study day were not significantly different, implying that participants had good compliance with consuming similar dietary intake leading up to each study day. Both the average of the three day food records and 24 hours prior to the study day were not different. Participants consumed approximately 2062 ± 74 calories a day and, as a percent of energy: 44% ± 1% as carbohydrate, 39% ± 4% as fat, and 21% ± 4% as protein. Saturated fat, sodium, and fiber intake were 11% ± 1% of calories, 3206 ± 316 mg, and 12 ± 1 g/1000 calories a day, respectively.

**Figure 2:**
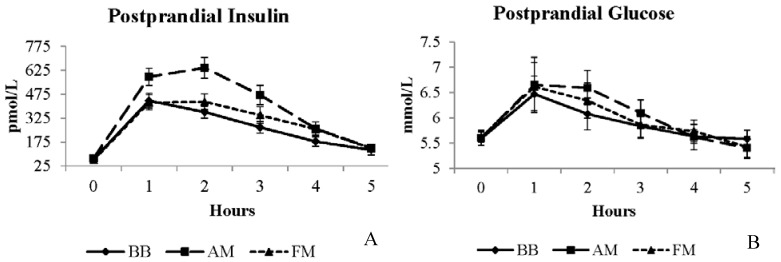
Postprandial insulin (**A**) and glucose (**B**) responses to moderate-fat breakfast with BB, AM, or FM. BB, black bean meal; FM, fiber matched meal; AM, antioxidant matched meal.

All meals resulted in elevated triglycerides over the five hour postprandial period, peaking around three hours and remaining elevated from baseline by five hours (time *p* < 0.0001) (Figure S1). No differences in the postprandial triglyceride responses were observed among meals (Table 3). No significant differences in glucose concentrations were observed among meals (Figure 2B and Table 3); however, a main effect of meal was apparent for the insulin response (meal *p* < 0.0001). The mean five hour insulin response after the BB meal was ~34% lower than the AM meal and the FM meal was ~24% lower than the AM meal (Figure 2A and Table 3).

**Table 2:** Baseline Labs (*n* = 12) ^a^.

Baseline Values	Units	Mean	SD
**Lipids**			
Triglycerides	mmol/L	1.5	0.7
Total-cholesterol	mmol/L	4.6	0.8
**Inflammation**			
hs-CRP ^b^	mg/dL	4.0	4.2
IL-6	pg/mL	2.2	1.3
IL-1β ^c^	pg/mL	ND	ND
sICAM1	ng/mL	202.5	3.1
sVCAM1	ng/mL	484.3	21.1
**Glycemia**			
Glucose	mmol/L	5.6	0.4
Insulin	pmol/L	68.2	25.1
HOMA-insulin resistance ^d^	ratio	2.4	1.0
HOMA-β cell function ^e^	%	92.6	32.0
Quantitative insulin sensitivity check index	ratio	0.34	0.02
Glucose:insulin ratio	ratio	11.5	2.8
**Oxidative stress**			
OxLDL	mU/L	49.8	11.4
Total ORAC ^f^	μmol/L trolox equivalents	18.6	12.7

hs-CRP, high sensitive C-reactive protein; ND, non-detectable; sICAM1, soluble intercellular adhesion molecule 1; sVCAM1, soluble vascular cell adhesion molecule 1; OxLDL, oxidized LDL; ORAC, oxygen radical absorbance capacity; HOMA, homeostasis model assessment. ^a^ Baseline labs were calculated as the mean of the baseline (fasting) values from each study day. ^b^ Only measured in the fasting state. ^c^ Despite being analyzed in two different labs by two different lab technicians, both fasting and postprandial results were non-detectable. The minimum detectable level was 0.03 pg/mL. ^d^ Normal HOMA-insulin resistance is 1 [15]. ^e^ Normal HOMA-β cell function is 100% [15]. ^f^ *n* = 11 for total ORAC.

**Table 3:** Postprandial Responses of Biochemical Measurements.

Biochemical Measurement	BB Meal LSM	FM Meal LSM	AM Meal LSM	SE	Units
Triglycerides ^b^	1.8	2.0	2.0	0.2	mmol/L
Glucose ^b^	5.9	5.9	6.0	0.2	mmol/L
Insulin ^a,b^	240.4	275.5	360.9 *	30.9	pmol/L
IL-6 ^b^	3.5	3.4	3.2	0.5	pg/mL
sICAM1	204.2	189.7 *	198.4	15	ng/mL
sVCAM1	492.7 *	449.5	458.6	30.9	ng/mL
Total ORAC ^a,b^	37.1	33.2 *	42.2	3.2	μmol/L TE
OxLDL	46.7	49.5	49.1	2.5	U/L

Values represent the least squares means (LSM) in the first three columns and the standard error (SE) in the fourth column, as an estimate of the five hour postprandial response after the BB, FM, and AM meals. LSM, least squares means; BB, black bean; FM, fiber matched; AM, antioxidant capacity matched; OxLDL, oxidized LDL; ORAC, oxygen radical absorbance capacity; sICAM1, soluble intercellular adhesion molecule 1; sVCAM1, soluble vascular cell adhesion molecule; TE, trolox equivalents 1 ^a^ indicates a significant meal × time interaction with * indicating the significant differences from pairwise comparisons. ^b^ indicates a significant time effect.

All meals resulted in elevated postprandial IL-6 over the five hour postprandial period (time *p* < 0.0001) (Figure S2). No differences in the postprandial IL-6 responses were observed among meals (Table 3). A main effect of meal was evident for the vascular inflammatory biomarkers, sICAM1 and sVCAM1 (*p* = 0.006 and *p* = 0.0002, respectively) (Table 3), although time and meal × time interactions were not different. The mean five hour postprandial sICAM1 response was ~7% higher after the BB meal than after the FM meal (*p* = 0.001) (Figure S3), an effect due to a gradual increasing trend after the BB meal and decreasing trend after the FM meal. The concentrations of sICAM1 were significantly different at the five hour time point between the BB and FM meals only (*p* = 0.002). The mean five hour sVCAM1 response was ~7% higher after the BB meal than after the AM meal (*p* = 0.002) and ~9% higher than after the FM meal (*p* < 0.0001) (Figure S4).

**Figure 3:**
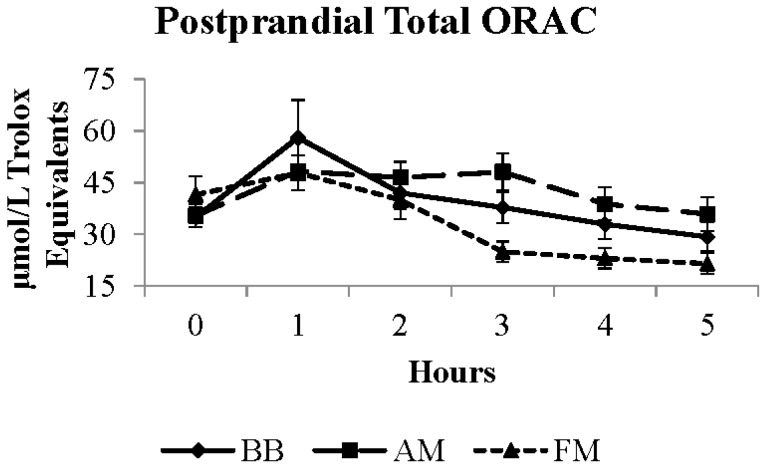
Postprandial total ORAC response to moderate-fat breakfast with BB, AM, or FM. BB, black bean meal; FM, fiber matched meal; AM, antioxidant matched meal; ORAC, oxygen radical absorbance capacity.

Oxidative status was analyzed by plasma total ORAC, a non-specific indicator of antioxidant capacity, and OxLDL, an indicator of oxidative damage. The postprandial total ORAC response was significant for meal (*p* < 0.0001), time (*p* < 0.0001), and meal × time interaction (*p* = 0.002). Overall, the AM meal produced the highest average five hour total ORAC value, which was ~21% higher than after the FM meal (*p* < 0.0001), which was ~11% higher than after the BB meal (*p* = 0.0004) (Figure 3). Despite meal related differences in plasma antioxidant capacity (total ORAC), no significant effects on OxLDL were apparent (Table 3).

## 4. Discussion

In the current study, inclusion of black beans into a typical Western meal favorably modified the postprandial (fed) insulin response in adults with MetS. Black bean in the meal (BB) resulted in an attenuated postprandial insulin response compared to meals controlling for the fiber fraction (FM) or the antioxidant capacity (AM) of black beans. These results did not directly translate to improvements in endothelial function markers or appear to influence or be due to changes in markers of inflammation or oxidative damage (OxLDL) in this study. Overall, the inclusion of black beans in a meal improved postprandial metabolic responses as indicated by postprandial insulin that could not be explained by either the fiber or antioxidant fractions alone.

Few studies have investigated the postprandial response to beans [11,12,13,14,15,16]. Thompson *et al.*, evaluated three bean and rice meals during a three hour postprandial period in adults with diabetes mellitus. The bean meals (comprised of black, pinto, and dark red kidney beans) attenuated postprandial glucose, compared to the rice control meal. Further, the two hour postprandial glucose response was < 140 mg/dL after the bean meals, which is consistent with diabetic glycemic control goals [16]. Other postprandial bean studies, investigating brown [12], white, and cream beans [13], and black bean extract [14], have also shown reduced glucose and insulin within a three hour postprandial period, compared to a reference food. In addition, white beans reduced postprandial lipemia, a result likely due to their different non-nutrient content (lower total galactoside, trypsin inhibitors, and lectin content) compared to cream beans [13]. Nilsson *et al.*, tested brown beans consumed as an evening test meal and measured two inflammatory biomarkers the next morning. IL-6 and IL-18 were reduced, which was explained, at least in part, by the actions of colonic fermentation [12]. In the current study, these effects on inflammatory biomarkers would not have occurred, as the meals would not have reached the colon in the postprandial period of five hours. In contrast to the aforementioned postprandial bean studies, others have not shown differences on postprandial glucose or insulin responses after consumption of pinto beans, navy beans, black-eyed peas (*Vigna unguiculata* subsp. *unguiculata*) [15], and white beans [11].

In the present study, we found significant effects of the BB meal on postprandial insulin responses, but not on glucose responses, although a trend was apparent in response to the BB meal. This may be explained in part by the fiber component of the black beans, since the meal containing equivalent amounts of fiber (FM) also reduced postprandial insulin compared to the AM meal, but not to the full extent of the BB meal. The anthocyanin content of the black beans could be playing a role in the improved insulin responses observed. Black beans provide a dietary source of total polyphenols, and specifically, anthocyanidins [28]. Dietary anthocyanins have been associated with improvements in insulin sensitivity and inflammation [29], which is consistent with clinical trial data using pure anthocyanins [30] or other dietary sources of anthocyanins [31]. The AM and BB meals were matched for antioxidant capacity, but purposely not for polyphenol content to be able to test the antioxidant property question. Collectively, the data support a role for specific effects of bioactive components, rather than a general antioxidant effect on outcomes related to insulin sensitivity. Whether the AM meal would have produced lower postprandial insulin or glucose responses, compared to a negative control lacking both fiber and antioxidant capacity, is unknown.

Vascular inflammation, as assessed by the adhesion molecules sICAM1 and/or sVCAM1, has been reported to decrease after postprandial dietary interventions, including antioxidant vitamins [32,33], walnuts [34], olive oil [34,35,36], and a high monounsaturated fat foods (spreads, cooking oils, baking fats, and mayonnaise) [37]. The findings from the current study do not support these findings directly, since time and meal × time interactions were not observed. A recent review found that adhesion molecules are not consistently altered postprandially, yet leukocyte bound markers are readily detected, which may be a better indicator of postprandial inflammation after high fat meals [38].

IL-6 concentrations increased after all meals, which aligns with some but not all studies: four out of eight studies have demonstrated an elevated postprandial IL-6 response to meals [3]. Postprandial inflammation is known to be sensitive to study design details, including length of postprandial assessments, meal challenge, or participants [39,40]. The current study collected blood samples out to five hours, whereas longer assessment periods of six or more hours may be necessary to measure intervention associated differences [39,40,41,42]. Choice of inflammatory markers may have also revealed different results. A high fat meal increased postprandial IL-18 and a high carbohydrate and high fiber meal (16.8 g) decreased the postprandial IL-18 response [43]. IL-18 is a relatively novel pro-inflammatory cytokine and recently associated with MetS [44] with an independent but modest relationship to cardiovascular disease [45]. Future studies will indeed want to expand inflammatory biomarkers to better understand postprandial inflammatory responses to dietary composition.

Several studies using different foods, including walnuts [46], pecans [47], antioxidant spice blend [48], mixed grape powder [49], mint herbs [50], blueberries [49,51,52], orange juice, orange flavonoids [53], kiwifruit, and cherries, have demonstrated that these additions to a typical meal enhance antioxidant defense, as measured by ORAC. Further, meals without these antioxidants have been shown to decline postprandial antioxidant capacity [49]. The results from the current study showed that the AM meal produced the highest ORAC value five hours postprandially, followed by the BB meal and then the FM meal. There was no significant influence of this enhanced antioxidant capacity on the endpoints measured. However, the last blood draw was obtained at five hours, which may have been too early to detect meal related differences in OxLDL. Examining the meal × time interaction curves of OxLDL clearly showed a declining trajectory of OxLDL after the AM and BB meals between four and five hours postprandially. Previous work has shown that differences in OxLDL between treatments may not be apparent until six hours postprandially [41,54]. Future research is needed to understanding the temporal dynamics of oxidative stress and damage to better discern the impact of dietary factors and to provide insight for developing dietary guidance.

This pilot study had limitations worth noting. First, the study did not include a negative control that contained no fiber and antioxidant capacity to fully evaluate the impact of the black beans or to show the potential benefits imparted by the individual dietary components. The AM provided 1259 μmol/L trolox equivalents more antioxidant capacity than the BB meal. However, the postprandial total ORAC response between the BB and AM meals was not significantly different and both were significantly elevated compared to the FM meal, so the overall difference likely did not favor the AM meal over the BB meal remarkably. Another limitation was that MetS has inherent heterogeneity in its definition [55]. However, MetS is increasing in incidence, so studying this at risk population provides potential applicability to a large portion of the population. Lastly, as a pilot study, enrolling one sex or increasing the sample size to circumvent potential sex differences was not feasible [3]. Some sex differences in adults with MetS have been reported in postprandial metabolic indices, such as women with lower triglycerides [56], men with more delayed triglyceride clearance [57], and women with elevated IL-6 [58].

## 5. Conclusions

Including black beans with a typical Western-style meal increases content of protein, dietary fiber, and an array of micronutrient and phytochemical components with many of the latter exhibiting the antioxidant property. After controlling for energy, protein, fiber, and antioxidant capacity in comparator meals, the meal with the black beans resulted in reduced postprandial insulin concentrations, suggesting enhanced insulin sensitivity in adults with MetS. These results support regular consumption of black beans with meals to manage postprandial metabolic responses, a strategy that would contribute to the metabolic health and delay of CVD and diabetes mellitus in adults with MetS.

## Supplementary Files

Supplementary File 1

## References

[1] Alberti K.G., Eckel R.H., Grundy S.M., Zimmet P.Z., Cleeman J.I., Donato K.A., Fruchart J.C., James W.P., Loria C.M., Smith S.C. (2009). Harmonizing the metabolic syndrome: A joint interim statement of the International Diabetes Federation Task Force on Epidemiology and Prevention; National Heart, Lung, and Blood Institute; American Heart Association; World Heart Federation; International Atherosclerosis Society; and International Association for the Study of Obesity. Circulation.

[2] Lutsey P.L., Steffen L.M., Stevens J. (2008). Dietary intake and the development of the metabolic syndrome: The Atherosclerosis Risk in Communities study. Circulation.

[3] Jackson K.G., Poppitt S.D., Minihane A.M. (2012). Postprandial lipemia and cardiovascular disease risk: Interrelationships between dietary, physiological and genetic determinants. Atherosclerosis.

[4] Bansal S., Buring J.E., Rifai N., Mora S., Sacks F.M., Ridker P.M. (2007). Fasting compared with nonfasting triglycerides and risk of cardiovascular events in women. JAMA.

[5] Nordestgaard B.G., Benn M., Schnohr P., Tybjaerg-Hansen A. (2007). Nonfasting triglycerides and risk of myocardial infarction, ischemic heart disease, and death in men and women. JAMA.

[6] Lindman A.S., Veierod M.B., Tverdal A., Pedersen J.I., Selmer R. (2010). Nonfasting triglycerides and risk of cardiovascular death in men and women from the Norwegian Counties Study. Eur. J. Epidemiol..

[7] Lairon D., Lopez-Miranda J., Williams C. (2007). Methodology for studying postprandial lipid metabolism. Eur. J. Clin. Nutr..

[8] Lairon D., Play B., Jourdheuil-Rahmani D. (2007). Digestible and indigestible carbohydrates: Interactions with postprandial lipid metabolism. J. Nutr. Biochem..

[9] Edirisinghe I., Randolph J., Cheema M., Tadapaneni R., Park E., Burton-Freeman B., Kappagoda T. (2012). Effect of grape seed extract on postprandial oxidative status and metabolic responses in men and women with the metabolic syndrome—Randomized, cross-over, placebo-controlled study. Funct. Foods Health Dis..

[10] Messina V. (2014). Nutritional and health benefits of dried beans. Am. J. Clin. Nutr..

[11] Bourdon I., Olson B., Backus R., Richter B.D., Davis P.A., Schneeman B.O. (2001). Beans, as a source of dietary fiber, increase cholecystokinin and apolipoprotein b48 response to test meals in men. J. Nutr..

[12] Nilsson A., Johansson E., Ekstrom L., Bjorck I. (2013). Effects of a brown beans evening meal on metabolic risk markers and appetite regulating hormones at a subsequent standardized breakfast: A randomized cross-over study. PLoS ONE.

[13] Olmedilla-Alonso B., Pedrosa M.M., Cuadrado C., Brito M., Asensio S.M.C., Asensio-Vegas C. (2013). Composition of two Spanish common dry beans (Phaseolus vulgaris), ‘Almonga’ and ‘Curruquilla’, and their postprandial effect in type 2 diabetics. J. Sci. Food Agric..

[14] Spadafranca A., Rinelli S., Riva A., Morazzoni P., Magni P., Bertoli S., Battezzati A. (2013). Phaseolus vulgaris extract affects glycometabolic and appetite control in healthy human subjects. Br. J. Nutr..

[15] Winham D.M., Hutchins A.M., Johnston C.S. (2007). Pinto bean consumption reduces biomarkers for heart disease risk. J. Am. Coll. Nutr..

[16] Thompson S.V., Winham D.M., Hutchins A.M. (2012). Bean and rice meals reduce postprandial glycemic response in adults with type 2 diabetes: A cross-over study. Nutr. J..

[17] Hutchins A.M., Winham D.M., Thompson S.V. (2012). Phaseolus beans: Impact on glycaemic response and chronic disease risk in human subjects. Br. J. Nutr..

[18] Bohn T. (2014). Dietary factors affecting polyphenol bioavailability. Nutr. Rev..

[19] Williamson G. (2013). Possible effects of dietary polyphenols on sugar absorption and digestion. Mol. Nutr. Food Res..

[20] Zhao Y., Du S.K., Wang H., Cai M. (2014). *In vitro* antioxidant activity of extracts from common legumes. Food Chem..

[21] Marathe S.A., Rajalakshmi V., Jamdar S.N., Sharma A. (2011). Comparative study on antioxidant activity of different varieties of commonly consumed legumes in India. Food Chem. Toxicol..

[22] USDA Oxygen Radical Absorbance Capacity (ORAC) of Selected Foods. http://www.ars.usda.gov/services/docs.htm?docid=15866.

[23] Go A.S., Mozaffarian D., Roger V.L., Benjamin E.J., Berry J.D., Blaha M.J., Dai S., Ford E.S., Fox C.S., Franco S. (2014). Heart disease and stroke statistics—2014 update: A report from the American Heart Association. Circulation.

[24] Prior R.L., Hoang H., Gu L., Wu X., Bacchiocca M., Howard L., Hampsch-Woodill M., Huang D., Ou B., Jacob R. (2003). Assays for hydrophilic and lipophilic antioxidant capacity (oxygen radical absorbance capacity (ORAC(FL))) of plasma and other biological and food samples. J. Agric. Food Chem..

[25] McCleary B.V., deVries J.W., Rader J.I., Cohen G., Prosky L., Mugford D.C., Okuma K. (2012). Determination of insoluble, soluble, and total dietary fiber (CODEX definition) by enzymatic-gravimetric method and liquid chromatography: Collaborative study. J. AOAC Int..

[26] Matthews D.R., Hosker J.P., Rudenski A.S., Naylor B.A., Treacher D.F., Turner R.C. (1985). Homeostasis model assessment: Insulin resistance and beta-cell function from fasting plasma glucose and insulin concentrations in man. Diabetologia.

[27] Katz A., Nambi S.S., Mather K., Baron A.D., Follmann D.A., Sullivan G., Quon M.J. (2000). Quantitative insulin sensitivity check index: A simple, accurate method for assessing insulin sensitivity in humans. J. Clin. Endocrinol. Metab..

[28] Wu X., Beecher G.R., Holden J.M., Haytowitz D.B., Gebhardt S.E., Prior R.L. (2006). Concentrations of anthocyanins in common foods in the United States and estimation of normal consumption. J. Agric. Food Chem..

[29] Jennings A., Welch A.A., Spector T., Macgregor A., Cassidy A. (2014). Intakes of anthocyanins and flavones are associated with biomarkers of insulin resistance and inflammation in women. J. Nutr..

[30] Li D., Zhang Y., Liu Y., Sun R., Xia M. (2015). Purified anthocyanin supplementation reduces dyslipidemia, enhances antioxidant capacity, and prevents insulin resistance in diabetic patients. J. Nutr..

[31] Stull A.J., Cash K.C., Johnson W.D., Champagne C.M., Cefalu W.T. (2010). Bioactives in blueberries improve insulin sensitivity in obese, insulin-resistant men and women. J. Nutr..

[32] Nappo F., Esposito K., Cioffi M., Giugliano G., Molinari A.M., Paolisso G., Marfella R., Giugliano D. (2002). Postprandial endothelial activation in healthy subjects and in type 2 diabetic patients: Role of fat and carbohydrate meals. J. Am. Coll. Cardiol..

[33] Neri S., Calvagno S., Mauceri B., Misseri M., Tsami A., Vecchio C., Mastrosimone G., Di Pino A., Maiorca D., Judica A. (2010). Effects of antioxidants on postprandial oxidative stress and endothelial dysfunction in subjects with impaired glucose tolerance and type 2 diabetes. Eur. J. Nutr..

[34] Cortes B., Nunez I., Cofan M., Gilabert R., Perez-Heras A., Casals E., Deulofeu R., Ros E. (2006). Acute effects of high-fat meals enriched with walnuts or olive oil on postprandial endothelial function. J. Am. Coll. Cardiol..

[35] Pacheco Y.M., Lopez S., Bermudez B., Abia R., Villar J., Muriana F.J. (2008). A meal rich in oleic acid beneficially modulates postprandial sICAM-1 and sVCAM-1 in normotensive and hypertensive hypertriglyceridemic subjects. J. Nutr. Biochem..

[36] Fuentes F., Lopez-Miranda J., Perez-Martinez P., Jimenez Y., Marin C., Gomez P., Fernandez J.M., Caballero J., Delgado-Lista J., Perez-Jimenez F. (2008). Chronic effects of a high-fat diet enriched with virgin olive oil and a low-fat diet enriched with alpha-linolenic acid on postprandial endothelial function in healthy men. Br. J. Nutr..

[37] Perez-Martinez P., Moreno-Conde M., Cruz-Teno C., Ruano J., Fuentes F., Delgado-Lista J., Garcia-Rios A., Marin C., Gomez-Luna M.J., Perez-Jimenez F. (2010). Dietary fat differentially influences regulatory endothelial function during the postprandial state in patients with metabolic syndrome: From the LIPGENE study. Atherosclerosis.

[38] Herieka M., Erridge C. (2014). High-fat meal induced postprandial inflammation. Mol. Nutr. Food Res..

[39] Devaraj S., Wang-Polagruto J., Polagruto J., Keen C.L., Jialal I. (2008). High-fat, energy-dense, fast-food-style breakfast results in an increase in oxidative stress in metabolic syndrome. Metabolism.

[40] Miglio C., Peluso I., Raguzzini A., Villano D.V., Cesqui E., Catasta G., Toti E., Serafini M. (2013). Antioxidant and inflammatory response following high-fat meal consumption in overweight subjects. Eur. J. Nutr..

[41] Burton-Freeman B., Talbot J., Park E., Krishnankutty S., Edirisinghe I. (2012). Protective activity of processed tomato products on postprandial oxidation and inflammation: A clinical trial in healthy weight men and women. Mol. Nutr. Food Res..

[42] Edirisinghe I., Banaszewski K., Cappozzo J., Sandhya K., Ellis C.L., Tadapaneni R., Kappagoda C.T., Burton-Freeman B.M. (2011). Strawberry anthocyanin and its association with postprandial inflammation and insulin. Br. J. Nutr..

[43] Esposito K., Nappo F., Giugliano F., di Palo C., Ciotola M., Barbieri M., Paolisso G., Giugliano D. (2003). Meal modulation of circulating interleukin 18 and adiponectin concentrations in healthy subjects and in patients with type 2 diabetes mellitus. Am. J. Clin. Nutr..

[44] Troseid M., Seljeflot I., Arnesen H. (2010). The role of interleukin-18 in the metabolic syndrome. Cardiovasc. Diabetol..

[45] Jefferis B.J., Papacosta O., Owen C.G., Wannamethee S.G., Humphries S.E., Woodward M., Lennon L.T., Thomson A., Welsh P., Rumley A. (2011). Interleukin 18 and coronary heart disease: Prospective study and systematic review. Atherosclerosis.

[46] Haddad E.H., Gaban-Chong N., Oda K., Sabate J. (2014). Effect of a walnut meal on postprandial oxidative stress and antioxidants in healthy individuals. Nutr. J..

[47] Hudthagosol C., Haddad E.H., McCarthy K., Wang P., Oda K., Sabate J. (2011). Pecans acutely increase plasma postprandial antioxidant capacity and catechins and decrease LDL oxidation in humans. J. Nutr..

[48] Skulas-Ray A.C., Kris-Etherton P.M., Teeter D.L., Chen C.Y., Vanden Heuvel J.P., West S.G. (2011). A high antioxidant spice blend attenuates postprandial insulin and triglyceride responses and increases some plasma measures of antioxidant activity in healthy, overweight men. J. Nutr..

[49] Prior R.L., Gu L., Wu X., Jacob R.A., Sotoudeh G., Kader A.A., Cook R.A. (2007). Plasma antioxidant capacity changes following a meal as a measure of the ability of a food to alter *in vivo* antioxidant status. J. Am. Coll. Nutr..

[50] Chusak C., Thilavech T., Adisakwattana S. (2014). Consumption of Mesona chinensis attenuates postprandial glucose and improves antioxidant status induced by a high carbohydrate meal in overweight subjects. Am. J. Chin. Med..

[51] Blacker B.C., Snyder S.M., Eggett D.L., Parker T.L. (2013). Consumption of blueberries with a high-carbohydrate, low-fat breakfast decreases postprandial serum markers of oxidation. Br. J. Nutr..

[52] Kay C.D., Holub B.J. (2002). The effect of wild blueberry (Vaccinium angustifolium) consumption on postprandial serum antioxidant status in human subjects. Br. J. Nutr..

[53] Snyder S.M., Reber J.D., Freeman B.L., Orgad K., Eggett D.L., Parker T.L. (2011). Controlling for sugar and ascorbic acid, a mixture of flavonoids matching navel oranges significantly increases human postprandial serum antioxidant capacity. Nutr. Res..

[54] Burton-Freeman B., Linares A., Hyson D., Kappagoda T. (2010). Strawberry modulates LDL oxidation and postprandial lipemia in response to high-fat meal in overweight hyperlipidemic men and women. J. Am. Coll. Nutr..

[55] Oda E. (2012). Metabolic syndrome: Its history, mechanisms, and limitations. Acta Diabetol..

[56] Cox-York K.A., Sharp T.A., Stotz S.A., Bessesen D.H., Pagliassotti M.J., Horton T.J. (2013). The effects of sex, metabolic syndrome and exercise on postprandial lipemia. Metabolism.

[57] Kolovou G.D., Anagnostopoulou K.K., Pavlidis A.N., Salpea K.D., Iraklianou S.A., Hoursalas I.S., Mikhailidis D.P., Cokkinos D.V. (2006). Metabolic syndrome and gender differences in postprandial lipaemia. Eur. J. Cardiovasc. Prev. Rehabil..

[58] Payette C., Blackburn P., Lamarche B., Tremblay A., Bergeron J., Lemieux I., Despres J.P., Couillard C. (2009). Sex differences in postprandial plasma tumor necrosis factor-alpha, interleukin-6, and C-reactive protein concentrations. Metabolism.

