# Supplementary material for: Black Beans, Fiber, and Antioxidant Capacity Pilot Study: Examination of Whole Foods vs. Functional Components on Postprandial Metabolic, Oxidative Stress, and Inflammation in Adults with Metabolic Syndrome
Source: Nutrients. 2015 Jul 27;7(8):6139–54. doi: 10.3390/nu7085273 (PMC4555112; doi:10.3390/nu7085273)
Supplement: Supplementary File 1 [file nutrients-07-05273-s001.docx]

**Supplementary Information**


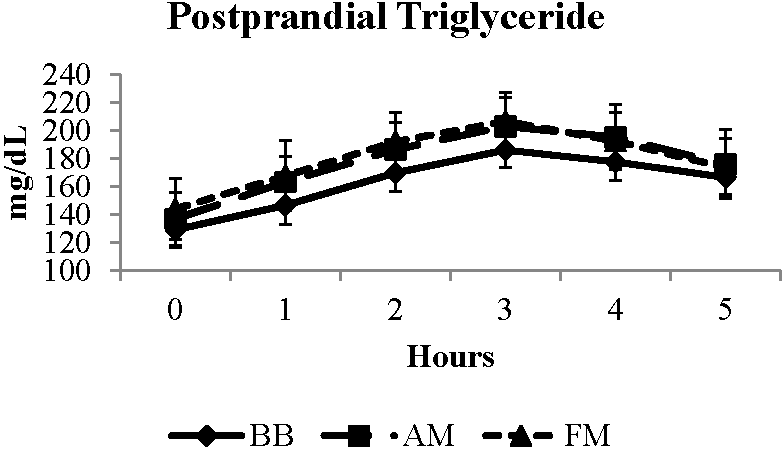


**Figure S1.** Postprandial triglyceride response to moderate-fat breakfast with BB, AM, or FM. BB, black bean meal; FM, fiber matched meal; AM, antioxidant matched meal; ORAC, oxygen radical absorbance capacity.


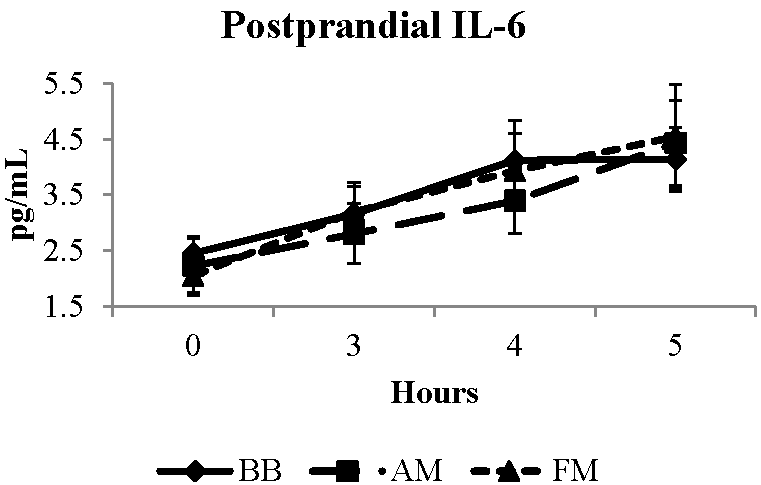


**Figure S2.** Postprandial IL-6 response to moderate-fat breakfast with BB, AM, or FM. BB, black bean meal; FM, fiber matched meal; AM, antioxidant matched meal; ORAC, oxygen radical absorbance capacity.


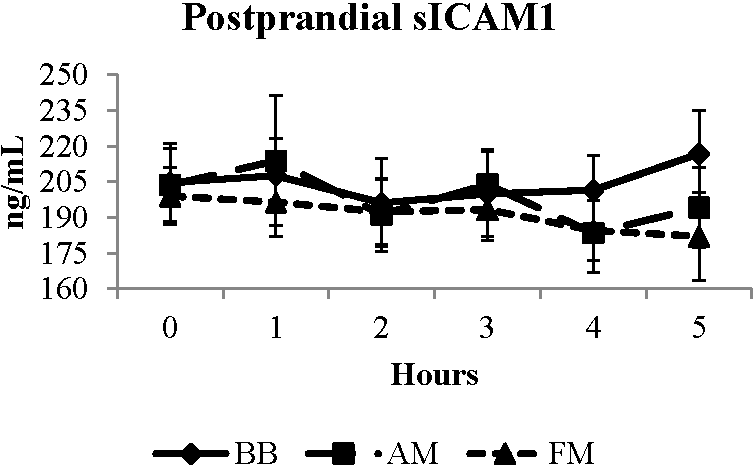


**Figure S3.** Postprandial sICAM1 response to moderate-fat breakfast with BB, AM, or FM. BB, black bean meal; FM, fiber matched meal; AM, antioxidant matched meal; ORAC, oxygen radical absorbance capacity.


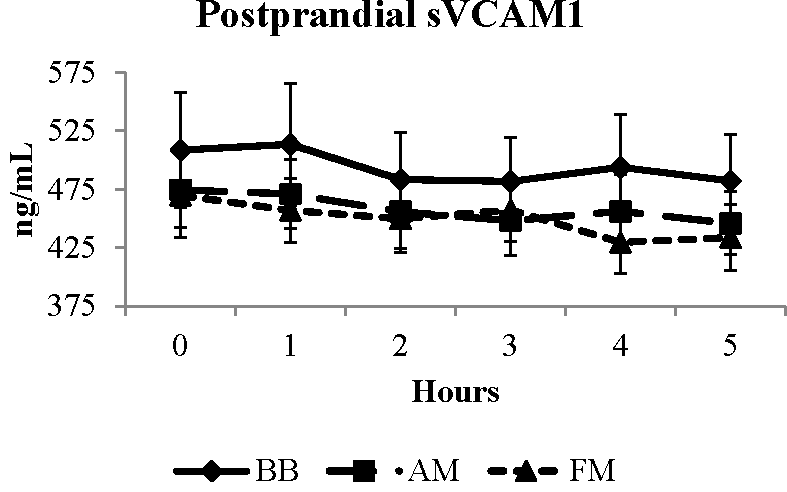


**Figure S4.** Postprandial sVCAM1 response to moderate-fat breakfast with BB, AM, or FM. BB, black bean meal; FM, fiber matched meal; AM, antioxidant matched meal; ORAC, oxygen radical absorbance capacity.

© 2015 by the authors; licensee MDPI, Basel, Switzerland. This article is an open access article distributed under the terms and conditions of the Creative Commons Attribution license (http://creativecommons.org/licenses/by/4.0/).
